# Supplementary figures and images for: JNK and p38 Inhibitors Prevent Transforming Growth Factor-β1-Induced Myofibroblast Transdifferentiation in Human Graves’ Orbital Fibroblasts
Source: Int J Mol Sci. 2021 Mar 14;22(6):2952. doi: 10.3390/ijms22062952 (PMC7998969; doi:10.3390/ijms22062952)

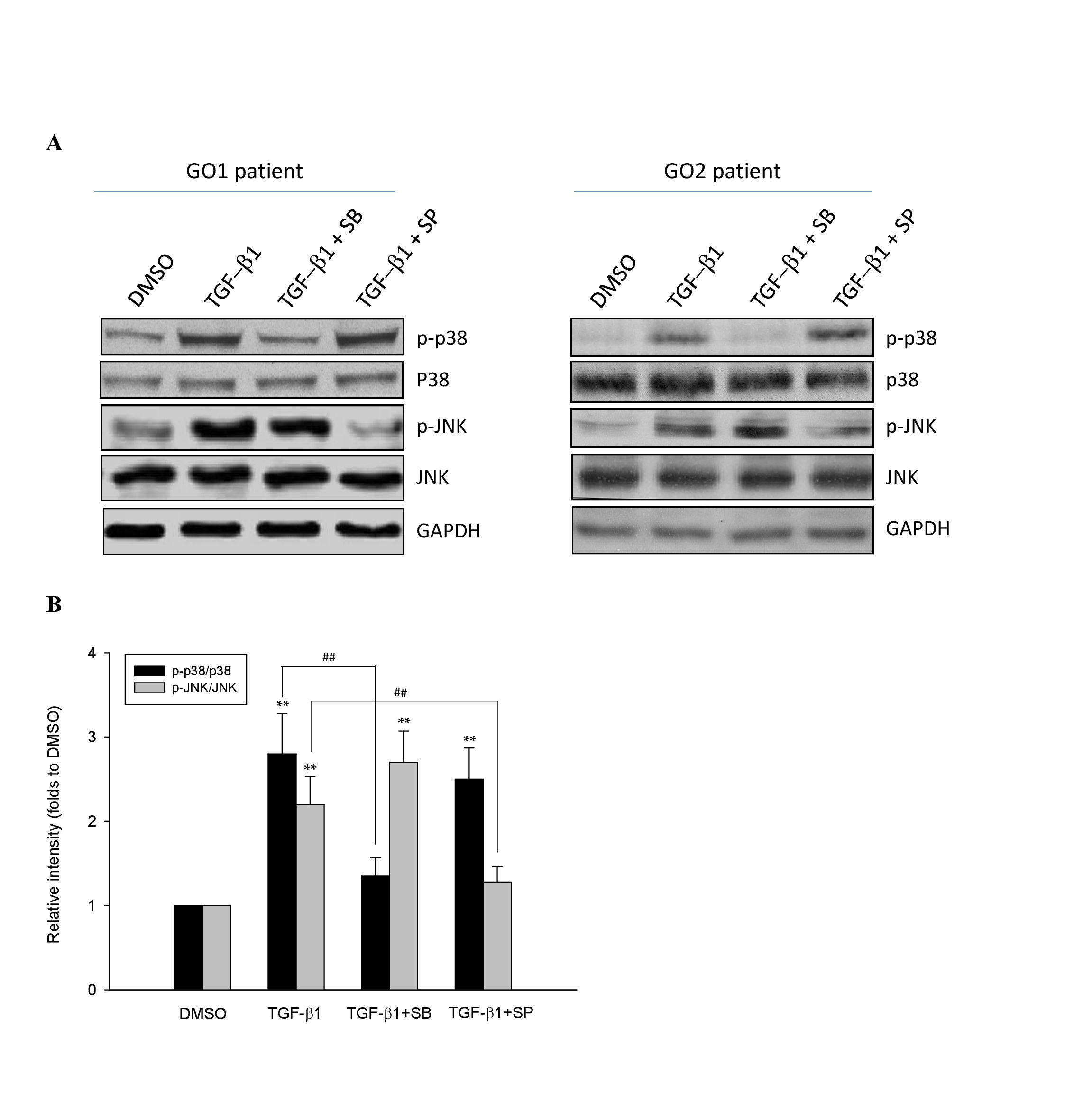

Supplement: Supplementary file 1 [file ijms-22-02952-s001.zip › Supplementary Materials/Figure S1.jpg]

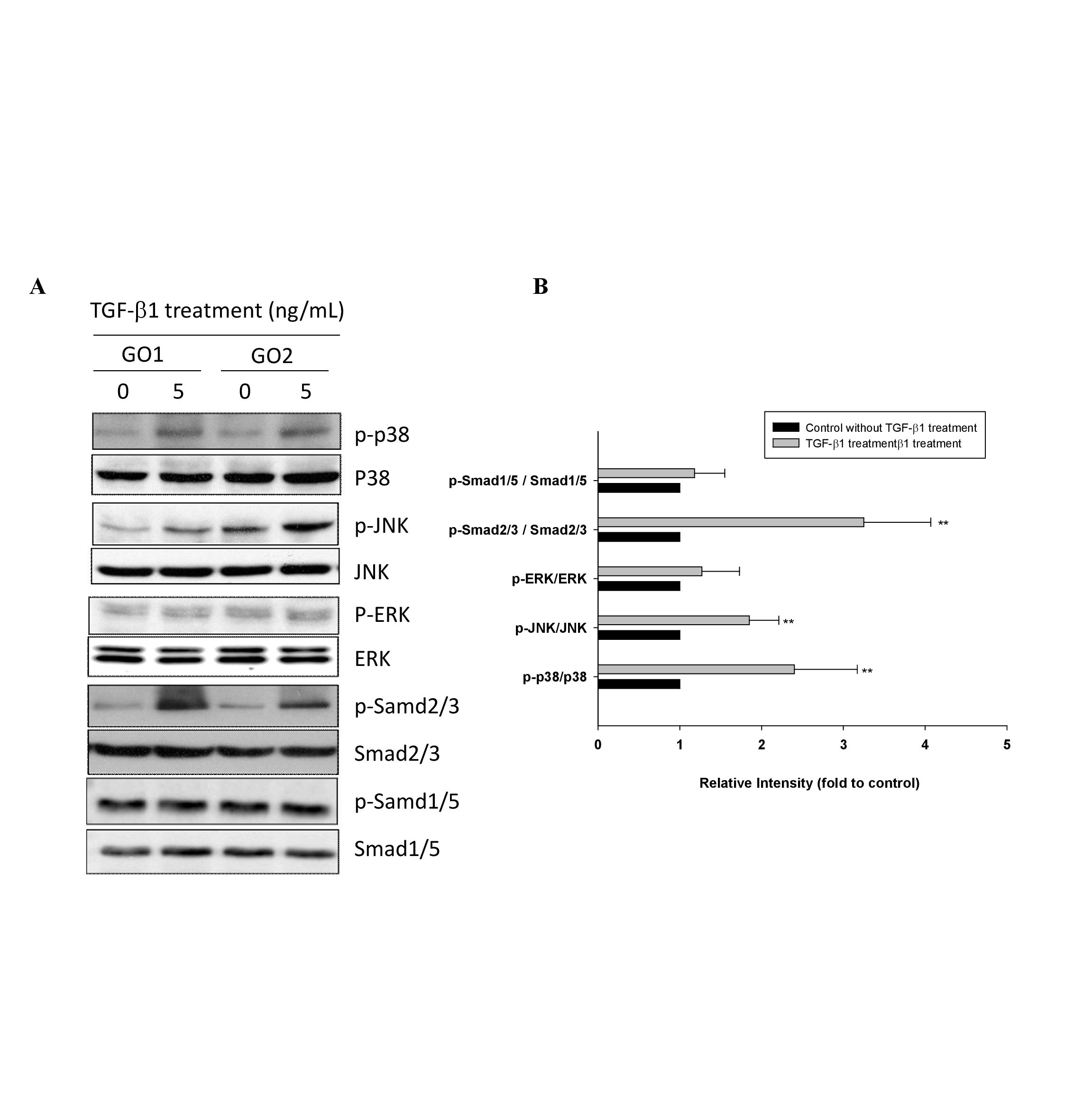

Supplement: Supplementary file 1 [file ijms-22-02952-s001.zip › Supplementary Materials/Figure S2.jpg]
